# Parasite detection and quantification in avian blood is dependent on storage medium and duration

**DOI:** 10.1002/ece3.9819

**Published:** 2023-02-09

**Authors:** Joshua G. Lynton‐Jenkins, Alexis S. Chaine, Andrew F. Russell, Camille Bonneaud

**Affiliations:** ^1^ Centre for Ecology and Conservation University of Exeter Penryn UK; ^2^ Station for Theoretical and Experimental Ecology CNRS Moulis France; ^3^ Institute for Advanced Studies in Toulouse Toulouse France

**Keywords:** avian malaria, DNA extraction, PCR, *plasmodium*, qPCR, Queen's lysis buffer

## Abstract

Studies of parasites in wild animal populations often rely on molecular methods to both detect and quantify infections. However, method accuracy is likely to be influenced by the sampling approach taken prior to nucleic acid extraction. Avian Haemosporidia are studied primarily through the screening of host blood, and a range of storage mediums are available for the short‐ to long‐term preservation of samples. Previous research has suggested that storage medium choice may impact the accuracy of PCR‐based parasite detection, however, this relationship has never been explicitly tested and may be exacerbated by the duration of sample storage. These considerations could also be especially critical for sensitive molecular methods used to quantify infection (qPCR). To test the effect of storage medium and duration on *Plasmodium* detection and quantification, we split blood samples collected from wild birds across three medium types (filter paper, Queen's lysis buffer, and 96% ethanol) and carried out DNA extractions at five time points (1, 6, 12, 24, and 36 months post‐sampling). First, we found variation in DNA yield obtained from blood samples dependent on their storage medium which had subsequent negative impacts on both detection and estimates of *Plasmodium* copy number. Second, we found that detection accuracy (incidence of true positives) was highest for filter‐paper‐stored samples (97%), while accuracy for ethanol and Queen's lysis buffer‐stored samples was influenced by either storage duration or extraction yield, respectively. Lastly, longer storage durations were associated with decreased copy number estimates across all storage mediums; equating to a 58% reduction between the first‐ and third‐year post‐sampling for lysis‐stored samples. These results raise questions regarding the utility of standardizing samples by dilution, while also illustrating the critical importance of considering storage approaches in studies of Haemosporidia comparing samples subjected to different storage regimes and/or stored for varying lengths of time.

## Graphical abstract

Studies of parasites in wild animal populations often rely on molecular methods to both detect and quantify infections from blood samples. We report that both blood storage medium and storage duration can impact the detection and quantification of *Plasmodium* parasites. This illustrates the importance of considering storage approaches in studies of Haemosporidia comparing samples subjected to different storage regimes and/or stored for varying lengths of time.

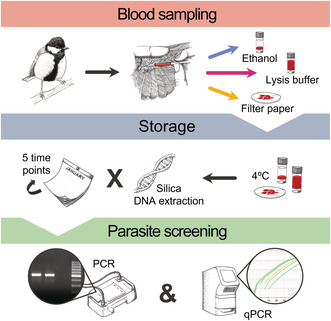

## 1 INTRODUCTION

Over the past three decades, ecological studies of host–parasites interactions have been greatly expanded by the advent of modern molecular techniques targeting nucleic acids for the detection, classification, and quantification of pathogens (Gasser, 1999; Watsa, 2020). This new toolkit has enhanced the detail attainable for each pathogen, opening the door to explorations that, historically, would have been unfeasible. However, the value of these insights only extends as far as the reliability of the data obtained, and due to the sensitivity of these molecular tools, it is essential that we consider how our methodological choices can bias results.

Molecular studies of disease in wild animal populations involve variations on a shared methodological pipeline: a sample (such as tissue, feces, or blood) is obtained from the animal and stored for future processing, nucleic acids are then isolated, before being analyzed using any of a variety of molecular techniques. Each step of this process has the potential to either directly or indirectly impact the accuracy of parasite detection in the population (i.e., prevalence estimates) and quantification within individuals (i.e., parasite load estimates). Within the medical and veterinary sciences, such considerations have resulted in the creation of best practice recommendations, where diagnostic guidance documents provide a strict framework allowing for cross‐study comparisons (Crobach et al., 2016; Toohey‐Kurth et al., 2020; WHO, 2019). However, ecological studies of disease in wild animals typically lack such guidance. Additional caution should, therefore, be taken where studies are collated with comparisons made between those applying different methodologies.

One field of study which has expanded rapidly following the advent of molecular detection techniques is that of avian blood parasites (Haemosporidia). These are insect vector protozoa encompassing the haemosporidian blood parasites related to those afflicting humans (e.g., genus *Plasmodium*). In birds, these parasites are studied both for their impact on hosts (e.g., from a conservation perspective) and because they provide a valuable host–parasite system within which to explore key questions in disease ecology and evolution (Bensch et al., 2009; Fecchio et al., 2019; LaPointe et al., 2012; Ricklefs et al., 2004). This has led to a considerable accumulation of knowledge, enabled, in part, by a range of molecular detection methods and quantitative PCR (qPCR) approaches, the use of which has become commonplace and widely accessible as new methodologies proliferate and reagent prices decline (Bell et al., 2015; Bensch et al., 2000; Friedl & Groscurth, 2012; Hellgren et al., 2004; Knowles et al., 2010; Schoener et al., 2017; Smith et al., 2015). However, while a number of authors have made methodological recommendations (Bensch et al., 2021), a wide range of methods are currently in use and no clear consensus has emerged on best practice approaches to sample processing.

In molecular studies of Haemosporidia using whole blood (i.e., the vast majority of studies), choosing which medium to use for sample storage is perhaps the first consequential decision prior to sampling any bird. Storage mediums include filter papers, lysis buffers, ethanol, EDTA buffers, and more, and choice can be limited by the practicalities of fieldwork conditions and access to long‐term storage space, but perhaps not made with parasite detection in mind. This is especially true when samples were originally collected for a different purpose (Owen, 2011), as DNA samples extracted for parentage analysis or population genetics projects have proven to be useful to many subsequent studies (e.g., molecular parasitology). An important consideration when making this choice is the evidence that storage medium can impact parasite detectability. For example, a within‐subject study of human blood infected by *Plasmodium* suggested that samples stored on filter paper were the most likely to show reductions in sensitivity depending on storage conditions (Färnert et al., 1999). Furthermore, a review by Freed and Cann (2006) on the storage of samples collected from birds highlighted apparent reduced accuracy in haemosporidian detection in laboratories that stored their samples in SDS lysis buffers, while those that used non‐lysing buffers did not, although variation in PCR methodology between laboratories may have also influenced detection. Reductions in detection sensitivity suggest that blood parasite quantification should also be influenced by storage medium choice, however, this question has not been directly addressed in studies of avian blood parasites. The impact of storage medium on blood sample stability may, in fact, be more significant for qPCR approaches than presence–absence detection by PCR. This is because, PCR approaches will be accurate if infection is above a minimum detection threshold (Bensch & Hellgren, 2020), while direct quantification by more sensitive qPCR approaches will incorporate any changes in parasite copy number resulting from the storage medium into erroneous estimates of parasite load. As sample storage mediums can vary even within a study (e.g., across time and sampling locations; Baillie & Brunton, 2011; Kulma et al., 2014; Schoener et al., 2020; Slowinski et al., 2018; Walther et al., 2014; Zehtindjiev et al., 2012), understanding the influence of storage medium on parasite detection and quantification is critical to robust studies of parasites and their hosts.

Beyond the choice of storage medium, an additional methodological consideration shared between all studies is the duration of sample storage prior to DNA extraction (Färnert et al., 1999; Permenter et al., 2015). This can vary vastly between studies, and yet storage duration prior to extraction typically goes unreported in sampling methodology (e.g., Knowles et al., 2011; Loiseau et al., 2013; Lynton‐Jenkins et al., 2020; van Rooyen et al., 2013). As a result, it is often unclear whether DNA was extracted rapidly after blood sampling or whether all extractions took place years later. And yet, there is good reason to believe storage duration could influence parasite quantification, as nucleic acids are known to degrade over time without optimal storage (Cannon et al., 2019; Huang et al., 2017; Sok et al., 2020). For example, in a study of *Mycoplasma genitalium* detection and quantification from urine samples stored for up to 5 weeks, quantification cycle (Cq) estimates increased with increasing storage duration prior to DNA extraction, indicating a decrease in *M. genitalium* DNA (Murray et al., 2019). As with medium choice, if storage duration prior to extraction impacts detectability or quantification, studies in which storage duration varies between comparison groups or in which time is not accounted for in analyses would risk false inferences.

**FIGURE 1:**
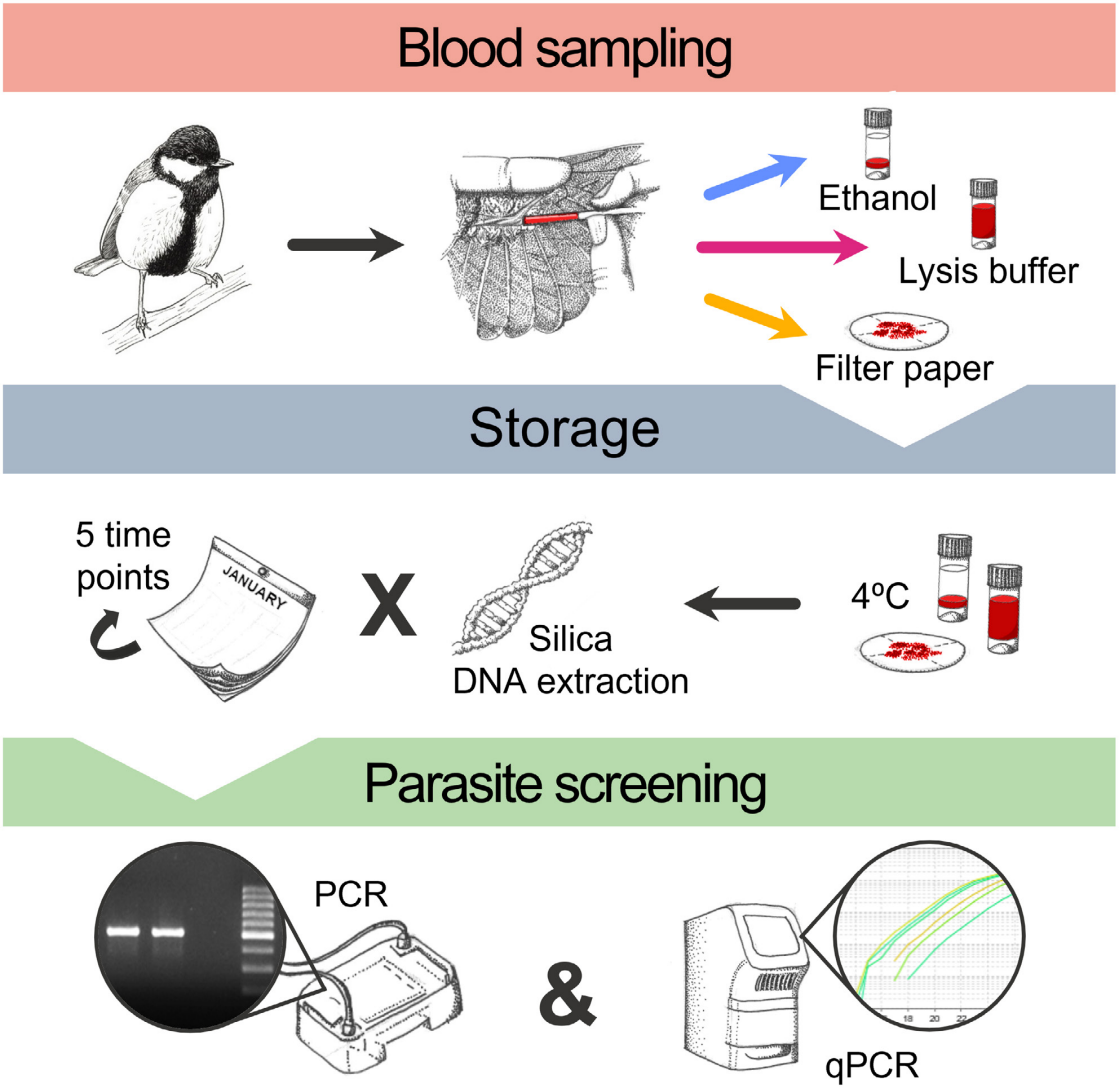
A graphical representation of the sampling process. Within‐individual samples were split between three storage mediums and stored at 4°C. DNA was extracted at five time points and parasite DNA was detected by PCR and qPCR.

In this study, we used a within‐individual experimental design to explore the impact of storage medium and storage duration on Haemosporidia detectability by PCR and quantification by qPCR. We made use of a host population of Paridae that are known to harbor high prevalence of avian blood parasites belonging to the genera *Haemoproteus* and *Plasmodium* (Lynton‐Jenkins et al., 2020). We obtained blood samples across a short capture window and split each sample into identical aliquots stored in three different storage mediums to compare PCR and qPCR efficiency when applied to DNA extracts obtained at five time points: 1, 6 months, 1, 2, and 3 years post‐capture. By making use of a widely used kit‐based extraction method and by storing all samples at a consistent temperature throughout the study, our aim was to specifically identify whether storage medium and storage duration would influence either parasite detection or parasite quantification in otherwise commonplace laboratory conditions (Figure 1).

## 2 MATERIALS AND METHODS

### 2.1 Sample collection and DNA extraction

Blood samples were collected from blue tits (*Cyanistes caeruleus*) and great tits (*Parus major*); a total of 24 individuals were caught using mist nets in the Pyrénées Ariégeoises Regional National Park, France (42°57′29″ N, 1°05′12″ E), between the 9th and 11th of November 2018. Samples were obtained by brachial venipuncture to a maximum volume of 75 μL from each individual and split approximately three ways: one part stored in 95% + ethanol (1 mL), one part in Queen's lysis buffer (1 mL) (Seutin et al., 1991), and one part on Whatman® filter paper (Grade 3). Hereafter, we refer to these storage mediums as ethanol, lysis buffer, and filter paper, respectively. All samples were then stored indefinitely at 4°C and subsamples were extracted at five time points: 1, 6 months, 1, 2, and 3 years post‐capture.

Total DNA was extracted from blood samples using a DNeasy Blood & Tissue extraction kit (QIAGEN®) following the manufacturer's protocol for nucleated blood. As DNA extraction methods vary, and techniques can be optimized within different lab groups (Tani et al., 2008), we used a kit‐based approach as these are increasingly used as one of the most accessible extraction methods. The digestion method varied dependent on the storage medium used, following QIAGEN suggested adjustments, and was kept consistent between time points, so too was the starting quantity of blood material used in the digestions. Therefore, both the digestion method and the exact volume of blood digested varied between the storage medium types, and caution should be applied in interpreting, e.g., differences in yields obtained dependent on storage medium. Full details of the extraction methods used are provided in the Supporting Information. Lastly, extraction quality and initial quantification were verified using a DropSense96™, and then more accurate quantification was obtained using a Qubit™ Fluorometer. Samples were then standardized to a working concentration of 25 ng/μL.

### 2.2 Sample screening

Samples were screened for blood parasite infections at each time point (within 2 days of extraction) using a nested polymerase chain reaction (PCR) method specific to *Plasmodium/Haemoproteus* (Lynton‐Jenkins et al., 2020). This method is a variation of well‐established Haemosporidian PCR detection techniques (Bensch et al., 2000; Hellgren et al., 2004), implemented here due to the high prevalence of *Leucocytozoon* parasites in these study populations. PCR products were resolved on 2% agarose gels stained with RedSafe™ Nucleic Acid Staining Solution (20,000x) (iNtRON Biotechnology Inc.) and run at 100 V for 60 min. All samples positive for *Plasmodium*/*Haemoproteus* parasite DNA were sequenced bi‐directionally using the primers HaemRP and HaemFP so that infection could be verified to genus level. Sequencing was performed by the Eurofins sequencing service (Eurofins‐MWG), processed using Geneious (Geneious® 9.1.5, Kearse et al., 2012), and parasites were identified via BLAST on the MalAvi database (Bensch et al., 2009). Individuals were classified as negative for infection when they either consistently presented no bands by PCR (*N* = 4) or if a faint band appeared, but no meaningful sequencing data were obtained in some screening runs (*N* = 2, 1 of 14 amplifications for one individual, and 2 of 13 amplifications for the other). Therefore, only individuals which were positive across more than two screening attempts and which when sequenced were verified as presenting parasite DNA were classified as positive (*N* = 17, all infections were of *Plasmodium relictum*). One sample was inconclusive, having presented apparent amplification in 42% (5/13) of screening attempts across all storage mediums, but with no confirmatory parasite sequence data obtained.

For samples positive for *Plasmodium*, we applied a qPCR approach (Knowles et al., 2010) to further qualify the degradation of parasite DNA as a function of storage medium and time since collection. Primers L9 5′‐AAACAATTCCTAACAAAACAGC‐3′ and NewR 5′‐ACATCCAATCCATAATAAAGCA‐3′ are used to amplify 188 bp of *Plasmodium* mitochondrial cytochrome b gene. To create standard curves, we amplified a 581 bp region of the *P. relictum* mitochondrial cytochrome b gene, encompassing the 188 bp target region of the L9/NewR primers, using primers RTL9 5’‐GGTAGCACTAATCCTTTAGGG‐3′ and RTNewR 5’‐CAGAAATGTCGTCTTATCGC‐3′. This 581 bp amplicon was ligated into a pCR™2.1 vector (The Original TA Cloning® Kit, Invitrogen™) and then transformed and cultured within One Shot™ TOP10 Chemically Competent E. coli (Invitrogen™). Plasmid DNA was isolated using a QIAprep® Spin Miniprep Kit (QIAGEN®) and quantified using a Qubit® dsDNA BR Assay (Thermo Fisher Scientific Inc.). DNA copy number was estimated using the molecular weight of the plasmid (including insert) and the quantified DNA concentration of the plasmid extract solution. From this solution, we created a dilution series to act as our standard curve; this series consisted of seven standards ranging from 4 to 4,021,503 *Plasmodium* DNA copies. Reactions were run on an Applied Biosystems™ StepOnePlus™ Real‐Time PCR system at volumes of 20 μL containing: 40 ng of DNA, qPCRBIO SyGreen Blue Mix Hi‐ROX, and 10 μM of each primer. The thermal profile consisted of a holding stage at 95°C for 20 seconds followed by amplification during 40 cycles of 95°C for 3 s and 60°C for 30 s. Reaction efficiency calculated from the standards using LinRegPCR v2021.1 (Ruijter et al., 2009) was found to be 99.8%. The repeatability of this approach has been previously validated (Knowles et al., 2010) and visual inspection of melt curves for each sample in our study confirmed the specific amplification of *Plasmodium*‐derived product. All samples were run in duplicate, and qPCR was conducted 3 years post‐collection, shortly after the extraction of samples that had been stored 3 years in the original collection medium and applied to samples extracted at 1 month, 2 years, and 3 years. After initial parasite screening (by PCR), all DNA extractions were stored frozen at −20°C until thawed for application of the qPCR. Therefore, although all DNA extractions experienced a similar freeze–thaw procedure, frozen storage duration varied between these three extraction time points. This is an important caveat to bear in mind and we discuss this limitation in the discussion to follow.

### 2.3 Statistical analyses

Statistical analyses were conducted using R version 3.6.3 (R Core Team, 2020). Parasite detection is likely to be impacted by the initial success of the DNA extraction. Therefore, to determine the effect of storage medium and storage duration on DNA extraction yields (ng/μL), a linear mixed model (LMM) was constructed using scaled log‐transformed yield as the response variable. In the maximal model, we fitted the interaction between storage medium (categorical) and storage duration (numerical: months passed post‐sampling) as a fixed effect. Individual ID (*N* = 24) was included as a random effect. We additionally explored the effect of storage medium and storage duration on DNA extraction quality. Using absorbance values obtained from the DropSense96™, we used the A_260_/A_280_ nm ratio to calculate Δ A_260_/A_280_, taking the absolute value by which each sample deviated from the ideal A_260_/A_280_ value of 1.80. Initially, an LMM was constructed using square root transformed Δ A_260_/A_280_ as the response variable. In the maximal model, we fitted the interaction between storage medium (categorical) and storage duration (numerical: months passed post‐sampling), and extraction yield (ng/μL) as fixed effects. Individual ID (*N* = 24) was included as a random effect, however, due to obtaining a singular fit with ID explaining little variance in the response term, we dropped ID and instead modeled the data using a linear model (LM) approach fitting the same fixed effects.

Second, to identify whether detection accuracy was dependent on storage medium and/or storage duration, we compared the rates of true positives obtained by PCR for those 17 individuals where infections had been verified as *P. relictum* (as described in 2.2 Sample screening). This was made possible as positive detection was not always obtained for these samples. Given that initial DNA extraction yield was significantly associated with storage medium, and to explore the storage medium‐specific effect of yield on detection accuracy, we modeled each medium independently (i.e., lysis and ethanol buffers only; filter‐paper‐stored samples were not modeled as they produced only two false negatives). We used a GLMM binomial regression (probit link function) modeling approach with successful or failed detection of a positive sample as the response variable, and with initial DNA yield and storage duration (numerical: months passed post‐sampling) and their interactions as a fixed effect. Individual ID (*N* = 17) was included as a random term.

Lastly, to determine the effect of storage medium and storage duration on parasite load (*Plasmodium* DNA copy number/μL DNA extract), we constructed LMMs using log‐transformed copy number as the response variable, and with initial DNA extraction yield, storage duration (months passed post‐sampling), and their interactions as fixed effects. As before, we modeled each storage medium separately to account for differences in extraction yield due to storage medium, and to explore the storage medium‐specific effect of yield on copy number. Individual ID (*N* = 14) was included as a random term due to the repeated measures of individuals. We excluded samples that produced more than one amplicon as indicated from melt curve analyses (*N* = 3); these samples were likely mixed infections and quantification was likely to be impacted. Finally, we compared variations in infection quantification across storage media. We calculated the coefficient of variation (CV) for measurements of copy number pertaining to each storage medium for each individual (i.e., pooling measures collected across time points). We then tested for differences in copy estimation consistency by applying a Kruskal–Wallis rank‐sum test to compare CV values between storage mediums and a post hoc Wilcoxon rank‐sum tests with Bonferroni adjustment to account for multiple testing.

All models were selected using the following approach; the dredge function (MuMIn package (Bartoń, 2019)) was used to select and rank models by their corrected Akaike information criteria (AICc) values and according to the model nesting rule (removing more complex models if a simpler nested version attained a lower AICc value (Harrison et al., 2018)). Top models were then selected as those with the lowest AIC scores, with a ΔAICc of at least 2 from the next model fit.

## 3 RESULTS

### 3.1 DNA extraction

**FIGURE 2:**
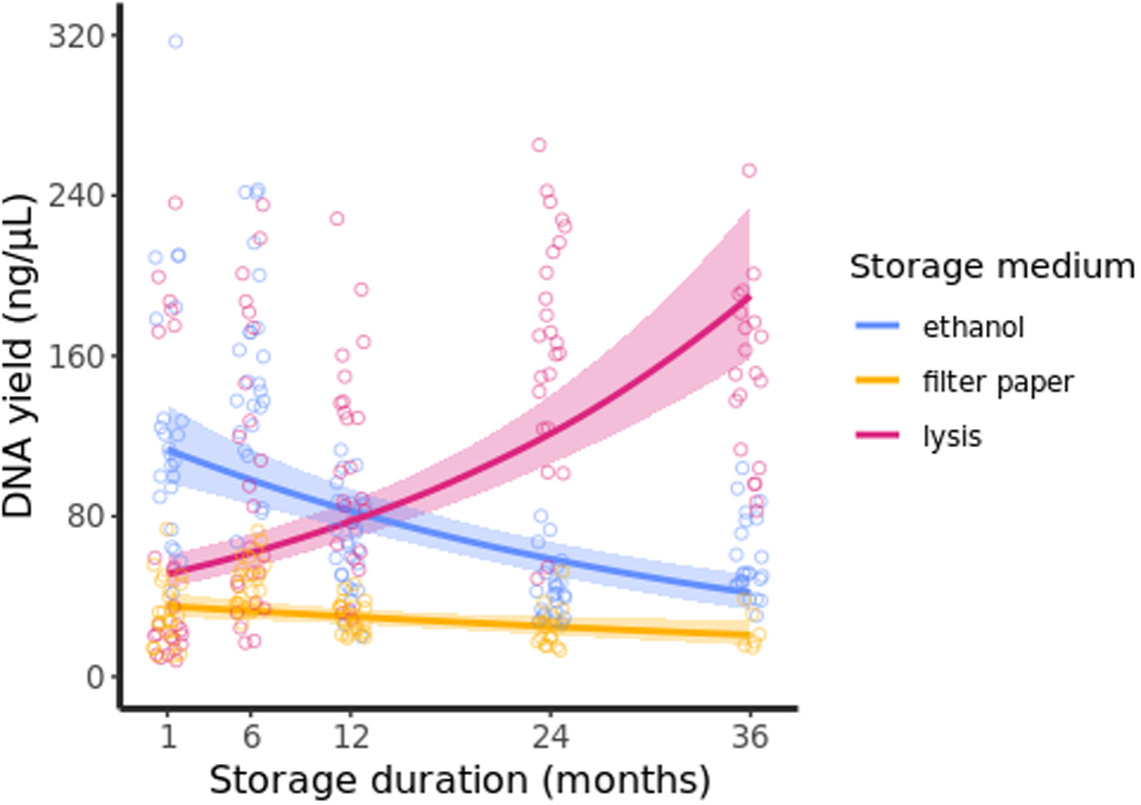
DNA yield of blood extractions as predicted for each storage medium dependent on storage duration. Plotted lines are predicted yield with shaded 95% confidence intervals. Distribution of the raw data is represented by points. Yield varied significantly across storage duration dependent on storage medium (Table 1).

Due to material limitations, not all individual sample–medium combinations were extracted across the later time points. This is because the amount of material available for extraction from filter paper was limiting and, as such, we were able to extract DNA for only 25% of individuals at the final 3 years post‐sampling time point (see Table S1 for sampling details). In contrast, the majority of samples were extracted at all five time points when stored in ethanol or lysis buffer. DNA extraction yield (quantified concentration of DNA in ng/μL) varied with storage duration, and this effect was storage medium dependent (Tables 1 and S2). DNA yield was highest for lysis‐stored samples (average yield ± SD: 117.0 ± 70.7) and second highest for those stored in ethanol (90.0 ± 57.4), while those on filter papers provided the lowest yields (33.2 ± 14.5). DNA yield increased for lysis‐stored samples with longer storage duration but decreased for samples stored in either ethanol or on filter papers (Figure 2). DNA quality (Δ A_260_/A_280_ from 1.8 optimum) varied with extraction yield and storage medium, being lowest in low‐yield samples and filter‐paper‐stored samples (Tables 2 and S3). Together, these results indicate that longer storage duration resulted in both increases and decreases in total DNA yields dependent on storage medium despite the application of a consistent extraction methodology. DNA quality was lowest for low‐yield extractions and, perhaps consequently, was lowest for filter‐paper‐stored samples (which may reflect the decrease in A_260_/A_280_ measurement accuracy at low nucleic acid concentrations).

**TABLE 1:** Top linear mixed model for tests of associations between DNA extraction yield in response to storage duration (months) and storage medium.

Covariate	Effect size	SE	95% CI	ΔAIC
DNA extraction yield (*N* = 330)				
Months	−0.04	0.005	−0.05 : −0.03	103.5*
Medium (Filter)	−1,46	0.15	−1.75 : −1.17	222.4*
Medium (Lysis)	−1.05	0.14	−1.33 : −0.77	“*
Months × Medium (Filter)	0.02	0.009	−0.0004 : 0.03	105.5
Months × Medium (Lysis)	0.08	0.007	0.07 : 0.1	“

Abbreviations: 95% CI, 95% confidence intervals; SE, standard error; ΔAIC, change in AIC with removal of covariate. The Model was selected using nested model approach for Δ4 AICc (full top model set: Table S2). Individual ID is included as a random effect (*N* = 24). Marginal/Conditional *R* ^2^ = .51 / .52. *Compared to the model including months × medium interaction term.

**TABLE 2:** Top linear model for tests of associations between DNA extraction quality (Δ A_260_/A_280_ nm) in response to extraction yield (ng/μL) and storage medium.

Covariate	Effect size	SE	95% CI	ΔAIC
DNA extraction quality (*N* = 330)				
Yield	−0.03	0.006	−0.04 : −0.01	15.03
Medium (Filter)	0.05	0.01	0.02 : 0.08	9.42
Medium (Lysis)	0.03	0.01	0.005 : 0.06	“

Abbreviations: 95% CI, 95% confidence intervals; SE, standard error; ΔAIC, change in AIC with removal of covariate. Model selected using nested model approach for Δ4 AICc (full top model set: Table S3). *R* ^2^ = .12.

### 3.2 Detection sensitivity

**FIGURE 3:**
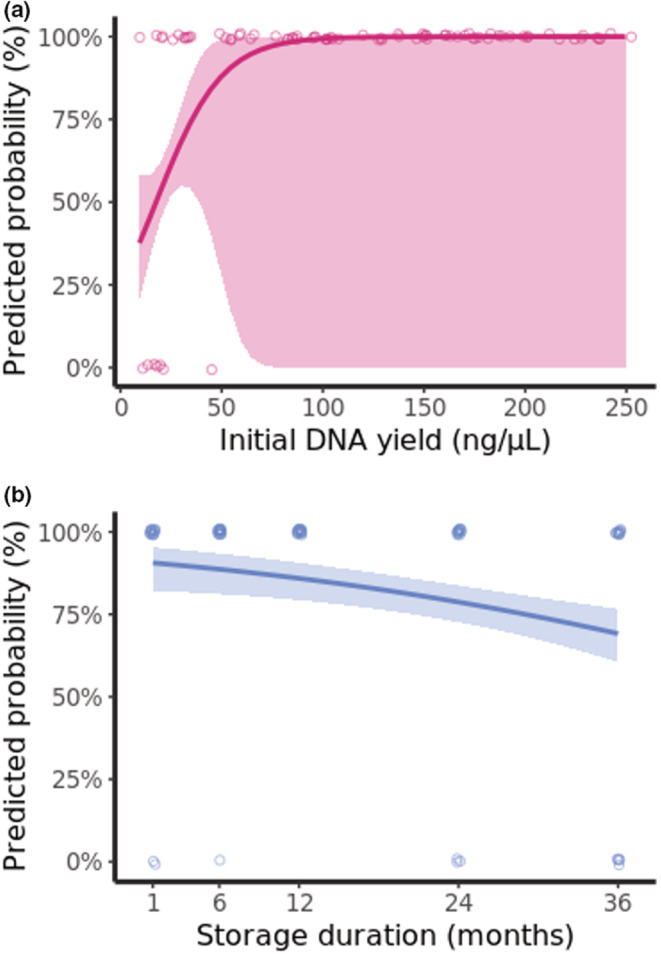
Predicted probability of accurate detection of *Plasmodium* by PCR for (a) lysis‐stored samples, where detection probability varied significantly dependent on initial DNA extraction yield (ng/μL), and (b) ethanol‐stored samples, where detection probability varied significantly dependent on storage duration. Plotted lines are predicted probability with shaded 95% confidence intervals (Table 2). Points are plotted raw data.

On the first occasion of screening at 1‐month post‐capture, prevalence ranged from 67 to 50% for *Plasmodium/Haemoproteus* infections depending on the storage medium, while the true prevalence was found to be 71% (*N* = 17, as described in 2.2 Sample Screening). All infections were identified as *P. relictum*. We estimate that detection accuracy (the number of positive amplifications of parasite DNA vs. false negatives) was the most consistent for filter‐paper‐stored samples, where only two false negatives occurred across all five time points. Therefore, for filter‐paper‐stored samples, overall detection accuracy was also the highest (97% across all screening occasions vs. 91% for lysis and 87% for ethanol‐stored samples). Meanwhile, detection accuracy of ethanol‐stored samples was found to decrease with storage duration, such that, between 1 and 3 years of storage, the probability of accurate detection decreased by 19% (Figure 3a; Tables 3 and S4). Detection accuracy for lysis‐stored samples was not influenced by storage duration but did vary with initial DNA extraction yield; indeed, the probability of accurate detection was found to increase by an average of 30% between yields of 25 and 50 ng/μL (Figure 3b; Tables 3 and S4). Prior to parasite screening, the DNA concentration of all samples was standardized to 25 ng/μL, so initial DNA extraction yield refers to the concentration obtained prior to dilution. In summary, while filter‐paper‐stored samples provided DNA extractions that allowed for the most accurate *Plasmodium* screening results; the accuracy of ethanol‐ and lysis‐stored samples was influenced by storage duration prior to extraction and initial extraction yield, respectively.

**TABLE 3:** Top models for tests of associations between detection accuracy in response to initial DNA extraction yield and storage duration (months).

Covariate	Effect size	SE	OR	95% CI	ΔAIC
1) Ethanol‐stored: accurate detection (*N* = 84)					
Months	−0.04	0.02	0.96	0.93 : 0.99	4.5
2) Lysis‐stored: accurate detection (*N* = 81)					
Yield	0.06	0.04	1.1	0.98 : 1.15	22.6

Abbreviations: 95% CI, 95% confidence intervals; SE, standard error; ΔAIC, change in AIC with removal of covariate. Storage mediums are modeled separately. Models were selected using nested model approach for Δ4 AICc (full top model set: Table S4). Individual ID was included as a random effect in all models (*N* = 17). Marginal/Condition Delta *R* ^2^ (Nakagawa & Schielzeth, 2013) = (1) .07/.25, (2) .84/.86.

### 3.3 Quantification of infections

**FIGURE 4:**
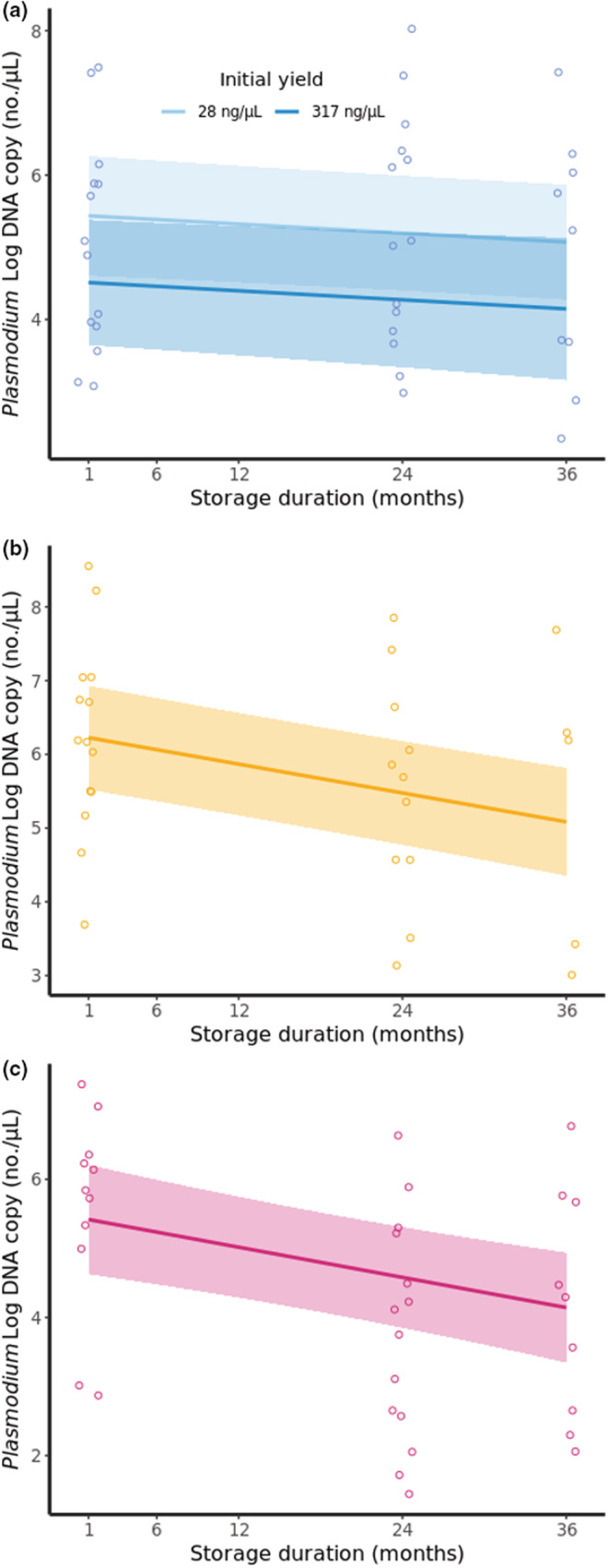
Predicted copy number of *Plasmodium* by qPCR for each storage medium dependent on storage duration; (a) ethanol‐stored; minimum and maximum of initial DNA extraction yields plotted to illustrate significant interaction, (b) filter‐paper‐stored, and (c) lysis‐stored; for averaged initial DNA extraction yield. Plotted lines are predicted copy numbers with shaded 95% confidence intervals. Raw data are points, two points removed in (c) at greater than 10,000 copies for clarity. Copy number estimates significantly decreased across storage duration for all storage mediums, and for ethanol‐stored samples, this decrease was dependent on extraction yield (Table 4).

**FIGURE 5:**
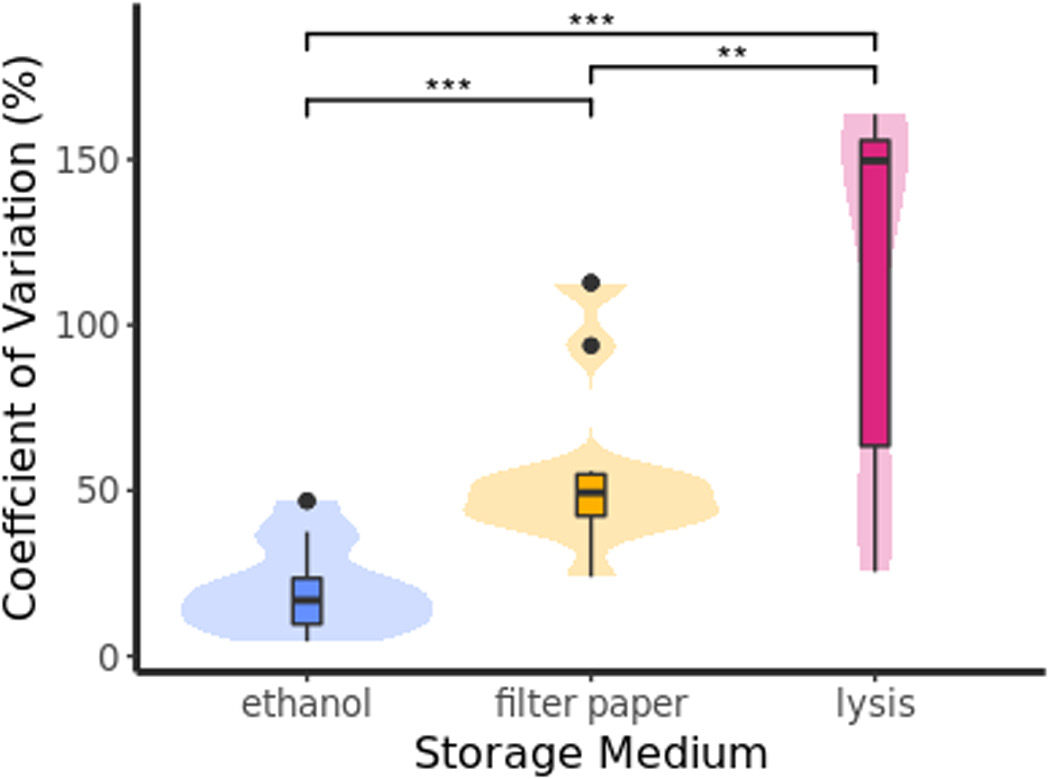
Coefficient of variation (CV) for within‐individual measurements of *Plasmodium* copy number plotted for each storage medium. Solid central lines of boxes represent the medium value, lower and upper box boundaries correspond to 1st and 3rd quartiles, and whiskers extend to the largest value ±1.5 × IQR. Outlying data are plotted as points and complete data are represented by shaded violins. Significance between groups is derived from post hoc Wilcoxon rank‐sum test; **p < .01; ***p < .001.

In all storage mediums, *Plasmodium* copy number decreased with longer storage duration prior to extraction (Tables 4 and S5). This reduction was highest for lysis‐stored samples, where predicted copy number decreased by 58% between the first and third year of storage (for average yield extractions) (Figure 4c). For filter‐paper‐stored samples, this decrease was 54% (Figure 4b), and for ethanol‐stored, it was 32%. However, in the case of ethanol‐stored samples, the reduction in copy number was dependent on an interaction with extraction yield, where high‐yield samples were associated with a greater reduction in copy number across storage duration, whereas lower‐yield samples showed little reduction (Figure 4a). Extraction yields also influenced copy number quantification in lysis‐stored samples, where those extracts with higher yields were found to have significantly lower copy numbers (Table 4). There was a significant difference in within‐individual measurement variation (as represented by CV) between the storage mediums (Kruskal–Wallis: *H*(2) = 62.2, *p* < .001). Extractions derived from ethanol‐stored blood produced the most consistent within‐individual estimates of copy number, and lysis‐stored blood produced the least (Figure 5). Storage duration was, therefore, found to decrease *Plasmodium* copy number estimates regardless of storage medium, although lysis‐stored samples presented both the largest decrease with longer storage and the least consistent estimates across repeated measures.

**TABLE 4:** Top models for tests of associations between *Plasmodium* copy number (*Plasmodium* DNA copy number/μL DNA extract) in response to initial DNA extraction yield and storage duration (months).

Covariate	Effect size	SE	95% CI	ΔAIC
1) Ethanol‐stored: copy number (*n* = 37)				
Yield	−0.003	0.001	−0.004 : −6.52	9.7*
Months	0.005	0.007	−0.01 : 1.91	6.8*
Yield × Months	−0.0003	0.001	−0.0005 : −0.00004	3.5
2) Filter‐paper‐stored: copy number (*n* = 30)				
Months	−0.03	0.005	−0.04 : −0.02	18.7
3) Lysis‐stored: copy number (*n* = 36)				
Yield	−0.01	0.002	−0.02 : −0.01	23.8
Months	−0.04	0.01	−0.06 : −0.02	8.8

Abbreviations: 95% CI, 95% confidence intervals; SE, standard error; ΔAIC, change in AIC with removal of covariate. Storage mediums are modeled separately. Models were selected using nested model approach for Δ4 AICc (full top model set: Table S5). Individual ID was included as a random effect in all models (*N* = 14). Marginal/Conditional *R* ^2^ = (1) .01 / .99, (2) .11 / .94, (3) .48 / .89. *Compared to model including yield × months interaction term.

## 4 DISCUSSION

In this study, we have explored how the storage of avian blood samples can influence Haemosporidia detection and quantification. Using blood collected from birds naturally infected with *P. relictum*, we compared the effects of both storage medium (96% ethanol, Queen's lysis buffer, and filter paper) and length of storage duration prior to DNA extraction. We found that storage medium influenced not only the DNA yields obtained from extractions but also detection accuracy (the rate of recording a true‐positive result) and parasite quantification. Meanwhile, increasing storage duration (up to 3 years post‐sampling) was found to be associated with both reduced detection accuracy for ethanol‐stored samples, and reductions in *Plasmodium* copy number estimates across all storage mediums. Copy number estimates obtained from lysis‐stored samples presented the greatest reduction with increased length of storage prior to extraction leading to the lowest consistency of *Plasmodium* copy number across samples. Taken together, these results highlight the fundamental importance of methodological considerations in long‐term comparative studies of avian Haemosporidia.

To obtain DNA extracts from blood samples stored using different preservative mediums, it is necessary to apply medium‐specific methodological approaches. These differences in extraction methodology are likely to influence the qualities of the final DNA extraction. Predictably, we found that blood stored using different mediums provided medium‐specific DNA yields. Similarly, and likely due to the unique composition of each storage medium, we detected medium‐specific changes in DNA yield with increasing storage duration. Perhaps the most surprising of these changes was the positive association between DNA yield and storage duration obtained from lysis‐stored samples, which most likely result from the specific chemistry of this storage medium (Seutin et al., 1991). The continued chemical action of the buffer (e.g., any change in pH) could be responsible for increased yields at later time points, as pH is an integral factor in silica‐based extraction methods (Griffiths & Chacon‐Cortes, 2014). By contrast, filter paper and ethanol are more chemically inert. These medium‐specific and temporal variations in yield, however, are only particularly noteworthy if they impact the results of downstream molecular analyses. Despite standardizing DNA extracts prior to the application of screening techniques, we found that extracts obtained from each medium did not produce comparable within‐sample measures of parasite load.

Estimates of parasite load, quantified here as *Plasmodium* copy number, should be obtainable from any high‐quality DNA extracts which are typically diluted to a standardized concentration prior to PCR. However, we found that lysis‐stored samples with high initial DNA extraction yield (prior to standardization) were associated with reduced *Plasmodium* copy number estimates. After accounting for storage duration, an increase in DNA yield between 50 and 100 ng/μL resulted in a halving of the estimated copy number. Dilution of initial DNA extractions is common in current avian Haemosporidia screening approaches (Bensch & Hellgren, 2020) as loading too much template DNA (which, for avian blood, is predominantly host DNA) can act as a PCR inhibitor (Cogswell et al., 1996). However, neither spectrophotometric nor fluorometric nucleic acid quantification methods can discern between host and parasite DNA in avian blood DNA extracts. It is, therefore, unknown whether higher‐concentration DNA extracts yield proportional increases in host and parasite DNA. This may not be the case if parasite DNA is initially more readily extracted from the sample, such that further increasing yields predominantly results in increases in host DNA. We speculate that this could explain the yield‐specific decreases in *Plasmodium* copy number detected here as high‐yield extracts require a higher dilution factor in order to be standardized. Future studies could resolve this discrepancy by comparing the stability of host and parasite DNA depending on storage approach in tandem. This would also determine whether sample standardization by dilution remains necessary, considering advances in *Taq* polymerase technologies which can perform well at much higher concentrations of DNA templates (Cating et al., 2012).

Beyond the effect of initial extraction yield, storage duration was also found to be associated with reduced *Plasmodium* copy estimation. As noted in our methodology, samples quantified from different time points were stored frozen for different lengths of time (earlier extractions were stored frozen for longer). If this variation in DNA storage was to impact parasite quantification, conventional wisdom would suggest that longer storage of frozen DNA could lead to degradation and decreased estimates of parasite copy number (Anchordoquy & Molina, 2007). We find the opposite pattern here (i.e., DNA samples extracted 1‐month post‐storage, and therefore, stored frozen for 2 years produced the highest estimates). This suggests that either the effect of frozen DNA storage duration was negligible or otherwise that the decrease in copy number estimates with increasing storage duration in the original collection medium is underestimated here and muted by the difference in DNA storage post‐extraction. Therefore, we find strong support for a negative effect of storage duration in the original collection medium on *Plasmodium* copy number estimation across all storage mediums. Furthermore, samples stored in lysis buffer showed the greatest variation in copy number estimation across the study, a result echoed in estimations of telomere length from samples stored in Queen's lysis buffer (Eastwood et al., 2018). One implication of this finding is that the rate of DNA degradation of parasite and host DNA may differ, or alternatively, that sample degradation disproportionally impacts the much lower concentration of parasite‐derived nucleic acids. This would introduce errors in estimating parasite load even in studies that quantify host DNA copy number alongside parasite copy number (e.g., Kulma et al., 2014). Copy number is used extensively in the quantification of diseases and sometimes used as a proxy for virulence (Mackinnon & Read, 2004; Garamszegi, 2006; but also see Tardy et al. (2019) on the limitations of this proxy). Accurate estimation is, therefore, essential and it is well established that caution should be applied in comparing copy number estimates between studies due to intra‐assay variation (Taylor et al., 2019). Here, we suggest that similar care should be taken in comparing between blood samples stored in different mediums within study, and between DNA extractions from samples collected across time, if they were not performed at a standardized time point post‐collection.

Degradation of genomic molecules is likely an inevitability of most contemporary blood sample storage methodologies (highlighting the value of detection by microscopy), but steps can be taken to avoid some of the consequences outlined in this study (Palmirotta et al., 2011; Schröder & Steimer, 2018). For example, while we used consistent extraction methods for each storage medium at each time point, it may be possible to partly compensate for medium‐specific effects by further optimizing extraction methodology to obtain higher yields (e.g., for ethanol‐stored samples 3 years post‐sampling). However, as noted, higher yields may only be of benefit if used in conjunction with a technique to avoid the dilution of parasite DNA relative to host DNA. Additionally, storage temperature is also likely to influence long‐term sample integrity (Cannon et al., 2019; Färnert et al., 1999; Richardson et al., 2006). Here, we kept samples refrigerated at 4°C, while long‐term storage at −20°C to −80°C may be preferable and could largely prevent DNA degradation (Chen et al., 2018; Färnert et al., 1999). Therefore, variations in extraction yields reported here are unlikely to directly apply to samples stored frozen. However, they are relevant to the many studies that do not use sub‐zero storage. Indeed, the benefit of some storage mediums is that they are thought to provide good sample preservation either at room temperature or chilled which allows for easier transport and storage location relative to frozen samples (Freed & Cann, 2006; Owen, 2011; Seutin et al., 1991). If such approaches are taken, considerations of how storage temperature effects may impact parasite screening should be made clear.

In conclusion, we have found that filter‐paper‐stored blood samples provided the most consistent results in terms of copy number estimation and detection accuracy of *Plasmodium* infections. High DNA extraction yields were obtained for lysis‐stored samples, but this did not necessarily improve parasite detection or copy number estimation, where yield and dilution effects may play a role in underestimation. Future efforts should be made to clarify the necessity of sample dilution prior to Haemosporidia screening. More generally, we found that *Plasmodium* copy number decreased with storage duration for all storage mediums tested. This effect could easily be misinterpreted in longitudinal studies if extracts are carried out simultaneously on stored samples of varying ages. In such cases, caution should be taken in interpreting results, suggesting increases in parasite load with time. Where temporal trends are of less interest, statistical approaches which account for storage duration prior to extraction may be sufficient to account for changes derived from laboratory processes. Our results also highlight the considerable within‐individual variation in parasite detection and quantification which can arise between extractions. While it is common for studies to include technical replicates, these typically make use of a single extraction and so consideration should be given instead to whether extraction replicates would better improve study accuracy. Ultimately, there are a great number of storage mediums available beyond the three explored in this study, but the choice of medium is often limited by practical considerations (Freed & Cann, 2006; Owen, 2011). Bearing this in mind, consistent sample storage and rigorous study design are the simplest measures that can be observed to ensure that the accurate characterization of infection is not obscured by artifacts of sampling methodology.

## AUTHOR CONTRIBUTIONS

**Joshua G. Lynton‐Jenkins:** Conceptualization (equal); data curation (lead); formal analysis (lead); investigation (lead); methodology (equal); writing – original draft (lead); writing – review and editing (supporting). **Alexis S. Chaine:** Conceptualization (supporting); funding acquisition (supporting); investigation (supporting); project administration (supporting); resources (equal); supervision (supporting); writing – review and editing (supporting). **A F Russell:** Funding acquisition (supporting); supervision (supporting); writing – review and editing (supporting). **Camille Bonneaud:** Conceptualization (equal); funding acquisition (lead); methodology (equal); project administration (lead); resources (lead); supervision (lead); writing – review and editing (lead).

## CONFLICT OF INTEREST STATEMENT

None declared.

## Supporting information


**Appendix S1.** Supporting Information

## Data Availability

Data supporting this study are available on Dryad: https://doi.org/10.5061/dryad.7h44j0zzd.
