# Supplementary material for: Parasite detection and quantification in avian blood is dependent on storage medium and duration
Source: Ecol Evol. 2023 Feb 9;13(2):e9819. doi: 10.1002/ece3.9819 (PMC9911630; doi:10.1002/ece3.9819)
Supplement: Supplementary file 1 — Appendix S1. Supporting Information [file ECE3-13-e9819-s001.docx]

Supporting Information for

Title: Parasite detection and quantification in avian blood is dependent on storage medium and duration.

Authors: *Lynton-Jenkins, Chaine, Russell, & Bonneaud*

**Contents**

- **Details of Extraction Methods**
- **Supporting Tables**
  - Table S1. Avian blood sample extraction counts and prevalence by storage medium.
  - Table S2. Top model set of LMM testing factors influencing DNA yield of extracts.
  - Table S3. Top model set of LM testing factors influencing DNA quality of extracts.
  - Table S4. Top model sets of GLMMs testing factors influencing correct *Plasmodium* detection by PCR.
  - Table S5. Top model sets of LMMs testing factors influencing *Plasmodium* copy number (*Plasmodium* DNA copy number/μL DNA extract).

**Details of Extraction Methods**

Samples were initially split three-ways and stored in either 95%+ ethanol, Queen’s Lysis buffer or on Whatman® filter paper (Grade 3). To perform extractions using DNeasy Blood & Tissue extraction kits (QIAGEN®) the protocol was adapted for each medium following QIAGEN recommendations and carried out consistently across time-points for each medium. Commercial buffers (AL, ATL, and AE) can be found in QIAGEN kits. All heated incubation steps were carried out on a Thermo Scientific™ Digital Heating Shaking Drybath. Once samples were digested, all remaining extraction steps (e.g., washes) followed the protocol as detailed for DNeasy Blood & Tissue extraction kits (QIAGEN®). Samples were eluted in 50µl of Buffer AE which was warmed to 30°C and added to the spin columns 30 minutes prior to elution by centrifugation. Medium-specific digestions steps were carried out as follows:

*Ethanol*

To start, 10µl of blood was obtained from the desiccated whole blood (which remains separate from the ethanol) and transferred to a microcentrifuge tube. Residual ethanol was evaporated by placing the 10µl sample in a dehydrator set to 30°C for 45 minutes. Added to this was 190µl of PBS, 200µl Buffer AL and 20µl Proteinase K. Samples were then incubated at 56°C for 180 minutes.

*Queen’s Lysis buffer*

To start, 200µl of sample was obtained from the homogenized diluted blood lysis solution and transferred to a microcentrifuge tube. This sample centrifuged at for 10 minutes and 100µl of the top fraction was removed. Added to the remaining 100µl was 100µl PBS, 200µl Buffer AL and 20µl Proteinase K. Samples were then incubated at 56°C for 120 minutes.

*Filter paper*

Three 3x3mm bloodspot cuttings were taken from the filter paper cards and transferred to a microcentrifuge tube. Bloodspots were digested initially in 180µl Buffer ATL for 10 minutes incubated at 85°C. Following this, 20µl of Protienase K was added and the sample incubated for a further 60 minutes at 56°C. Finally, 200µl of Buffer AL was added with a final incubation stage at 70°C for 10 minutes.

**Table S1.** Table of avian blood sample extraction counts and screening results for each storage medium, extractions carried out across increasing storage duration post-sampling. Initially, 24 individuals were sampled and reductions in sample sizes reflect cases where insufficient sample existed for an individual’s continued inclusion in the study. Sample size (N) is given as the number of samples included per medium and timepoint / the number of those samples considered true positives (defined in methods) / the number of those true positive samples which tested positive in that instance. Accuracy is given as the percentage of true positives correctly identified at a given timepoint per medium.

| **Storage duration** | | **Storage medium** | | |
| --- | --- | --- | --- | --- |
| **months** | | **ethanol** | **filter** | **lysis** |
| **1** | N: | 24/17/15 | 24/17/16 | 24/17/12 |
|  | Accuracy: | 88% | 94% | 50% |
| **6** | N: | 24/17/16 | 24/17/17 | 24/17/15 |
|  | Accuracy: | 94% | 100% | 88% |
| **12** | N: | 24/17/17 | 23/16/16 | 24/17/17 |
|  | Accuracy: | 100% | 100% | 100% |
| **24** | N: | 24/17/14 | 18/13/12 | 24/17/17 |
|  | Accuracy: | 82% | 92% | 100% |
| **36** | N: | 23/16/11 | 6/6/6 | 20/13/13 |
|  | Accuracy: | 69% | 100% | 100% |
|  |  |  |  |  |

**Table S2.** Top model set of LMM testing factors influencing DNA yield of extracts. Individual ID included as random effect (N = 24). Produced using nested model approach for Δ4 AICc. df = degrees of freedom; ΔAICc = change in AICc from the lowest AICc; Weight = Adjusted Akaike model weight.

| Covariates | Intercept | df | logLik | AICc | ΔAICc | Weight |
| --- | --- | --- | --- | --- | --- | --- |
| **DNA Yield** (n = 330) | | | | | | |
| Month * Medium | 0.8051 | 8 | -351.7 | 719.9 | 0 | 1 |

**Table S3.** Top model set of LM testing factors influencing DNA quality of extracts. Produced using nested model approach for Δ4 AICc. df = degrees of freedom; ΔAICc = change in AICc from the lowest AICc; Weight = Adjusted Akaike model weight.

| Covariates | Intercept | df | logLik | AICc | ΔAICc | Weight |
| --- | --- | --- | --- | --- | --- | --- |
| **DNA quality** (n = 330) | | | | | | |
| Month * Medium | 0.8051 | 8 | -351.7 | 719.9 | 0 | 1 |

**Table S4.** Top model sets of GLMMs testing factors influencing correct *Plasmodium* detection by PCR. Individual ID included as random effect (N = 17). Produced using nested model approach for Δ4 AICc. df = degrees of freedom; ΔAICc = change in AICc from the lowest AICc; Weight = Adjusted Akaike model weight.

| Covariates | Intercept | df | logLik | AICc | ΔAICc | Weight |
| --- | --- | --- | --- | --- | --- | --- |
| **Ethanol stored: accurate detection** (n = 84) | | | | | | |
| Month | 2.951 | 3 | -28.145 | 62.6 | 0 | 0.874 |
| Intercept | 2.439 | 2 | -31.157 | 66.5 | 3.87 | 0.126 |
| **Lysis stored: accurate detection** (n = 81) | | | | | | |
| Yield | 54.96 | 3 | -10.1 | 26.4 | 0 | 1 |

**Table S5.** Top model sets of LMMs testing factors influencing *Plasmodium* copy number (*Plasmodium* DNA copy number/μL DNA extract). Storage mediums modelled separately. Individual ID included as random effect (N = 14). Produced using nested model approach for Δ4 AICc. df = degrees of freedom; ΔAICc = change in AICc from the lowest AICc; Weight = Adjusted Akaike model weight.

| Covariates | Intercept | df | logLik | AICc | ΔAICc | Weight |
| --- | --- | --- | --- | --- | --- | --- |
| **Ethanol stored: copy number** (n = 37) | | | | | | |
| Yield * Month | 5.46 | 6 | -25.867 | 66.5 | 0 | 0.78 |
| Yield + Month | 5.53 | 5 | -28.587 | 69.1 | 2.57 | 0.22 |
| **Filter stored: copy number** (n = 30) | | | | | | |
| Month | 6.26 | 4 | -34.427 | 78.5 | 0 | 1 |
| **Lysis stored: copy number** (n = 36) | | | | | | |
| Yield + Month | 7.38 | 5 | -52.359 | 116.7 | 0 | 1 |
